# Effect of electro-acupuncture on ovarian expression of α (1)- and β (2)-adrenoceptors, and p75 neurotrophin receptors in rats with steroid-induced polycystic ovaries

**DOI:** 10.1186/1477-7827-3-21

**Published:** 2005-06-07

**Authors:** Luigi Manni, Thomas Lundeberg, Agneta Holmäng, Luigi Aloe, Elisabet Stener-Victorin

**Affiliations:** 1 Cardiovascular Institute and Wallenberg Laboratory, Sahlgrenska Academy, Göteborg University, SE-413 45 Göteborg, Sweden; 2 Institute of Neurobiology and Molecular Medicine (CNR), Rome, Italy; 3 Rehabilitation Medicine, Karolinska Hospital, SE-171 77 Stockholm, Sweden; 4 Department of Obstetrics and Gynaecology, Sahlgrenska University Hospital, Sahlgrenska, SE-413 45 Göteborg, Sweden; 5 Institute of Occupational Therapy and Physical Therapy, Sahlgrenska Academy, Göteborg University, SE-405 30 Göteborg, Sweden

## Abstract

**Background:**

Estradiol valerate (EV)-induced polycystic ovaries (PCO) in rats is associated with an increase in ovarian sympathetic outflow. Low-frequency (2 Hz) electro-acupuncture (EA) has been shown to modulate sympathetic markers as well as ovarian blood flow as a reflex response via the ovarian sympathetic nerves, in rats with EV-induced PCO.

**Methods:**

In the present study, we further tested the hypothesis that repeated 2 Hz EA treatments modulate ovarian sympathetic outflow in rats with PCO, induced by a single i.m. injection of EV, by investigating the mRNA expression, the amount and distribution of proteins of α1a-, α1b-, α1d-, and β2-adrenoceptors (ARs), as well as the low-affinity neurotrophin receptor (p75NTR).

**Results:**

It was found that EV injection results in significantly higher mRNA expression of ovarian α1b- and α1d-AR in PCO rats compared to control rats. The p75NTR and β2-ARs mRNA expression were unchanged in the PCO ovary. Low-frequency EA resulted in a significantly lower expression of β2-ARs mRNA expression in PCO rats. The p75NTR mRNA was unaffected in both PCO and control rats. PCO ovaries displayed significantly higher amount of protein of α1a-, α1b- and α1d-ARs, and of p75NTR, compared to control rats, that were all counteracted by repeated low-frequency EA treatments, except for α1b-AR.

**Conclusion:**

The present study shows that EA normalizes most of the EV-induced changes in ovarian ARs. Furthermore, EA was able to prevent the EV-induced up regulation of p75NTR, probably by normalizing the sympathetic ovarian response to NGF action. Our data indicate a possible role of EA in the regulation of ovarian responsiveness to sympathetic inputs and depict a possible complementary therapeutic approach to overcoming sympathetic-related anovulation in women with PCOS.

## Introduction

Polycystic ovary syndrome (PCOS) is a heterogeneous endocrine and metabolic disorder recognized as the primary cause of infertility in women of the reproductive age [1]. The syndrome is associated with ovulatory dysfunction, abdominal obesity, hyperandrogenism, and profound insulin resistance [1].

The precise etiology of the disease is unknown, even though the disturbances detected in PCOS has been attributed to primary defects in the hypothalamus-pituitary-adrenal (HPA) axis, the ovarian microenvironment, the adrenal gland, and the insulin/insulin-like growth factor (IGF)-I metabolic regulatory system [1]. That the sympathetic nervous system may be a primary factor in the development and maintenance of PCOS has been suggested by several investigators [2-5].

The utility of murine models of polycystic ovaries (PCO) has been discussed [6]. Even though it is impossible to reproduce human PCOS in an animal model, such a model may provide important leads. Studies on adult normal cycling rats found that a single intramuscular (i.m.) injection of estradiol valerate (EV) causes acyclicity and formation of PCO [7]. The EV-induced rat PCO model reflects some endocrinological and morphological characteristics of human PCOS, and it is assumed that activity in the ovarian sympathetic nerves is higher than in normal rats [8-10]. This is evidenced by an early increase in ovarian levels of norepinephrine (NE), an enhanced release of NE from ovarian nerve terminals, an increased activity of the catecholamine synthesis-limiting enzyme tyrosine hydroxylase (TH), and down-regulation of β_2_-adrenoceptors (ARs) in theca-interstitial cells [8-10].

The expression of other types of ARs in the ovary, namely the α_1_-ARs, has been evaluated by functional studies. α_1_-ARs are members of the G protein-coupled receptors and play critical roles in the regulation of a variety of physiological processes [11]. Within this classification, there are three subtypes: α_1a_, α_1b_, and α_1d _[11]. The α_1a_-AR subtype has been reported to be implicated in the maintenance of vascular basal tone, the α_1b_-AR subtypes to participate in the response to exogenous agonists, and that the α_1d_-AR subtype is a predominant mediator of arterial vasoconstriction. In vitro studies have demonstrated that α-AR are involved in the regulation of ovarian blood flow [12] and most probably in the ovarian steroidogenesis [13]. In a recent study, we found that the expression of all the α_1_-AR subtypes in the ovaries of PCO rats significantly differs from that of controls and varies at different time points after EV injection, indicating a possible participation of this ARs in the development of EV-induced PCO [14].

It has been demonstrated that the development of ovarian follicular cysts in steroid-induced PCO in rats is preceded by an increased synthesis of ovarian nerve growth factor (NGF) and low-affinity neurotrophin receptor (p75^NTR^) mRNA [10]. Thus, blocking the actions of intra-ovarian NGF restores estrus cyclicity as well as structural and functional features of the ovary in EV-induced PCO in rats [10], suggesting that hyper activation of sympathetic input in PCO is related to an overproduction of NGF.

Electro-acupuncture (EA) is a non-pharmacological method known to initiate a number of reactions at the spinal level and centrally in the brain [15,16]. We have recently demonstrated that repeated low-frequency EA treatments induced regular ovulations in more than one-third of the women affected by PCOS and normalized endocrine and neuroendocrine parameters without any negative side-effects [17]. These observation suggest that EA effects are mediated through inhibition of the activity of the sympathetic nervous system since EA is known to modulate various autonomic functions [17]. Moreover, using the steroid-induced PCO model, we found that repeated treatments of low-frequency EA in somatic segment related to the innervation of the ovary, reduced high concentrations of ovarian NGF, corticotrophin-releasing factor (CRF), and endothelin-1 as well as increased low concentrations of hypothalamic β-endorphin [18-21]. Furthermore, low-frequency EA increases ovarian blood flow as a reflex response via the ovarian sympathetic nerves, whereas high frequency decreases ovarian blood flow as a passive response following systemic circulatory changes in both normal and PCO rats [22,23]. These results suggest that repeated treatments of low-frequency, but not high frequency EA, can inhibit high activity in the autonomic nervous system. However, the mechanism implicated in this event is not clearly known.

The present study was undertaken to investigate the effect of repeated treatments of low-frequency EA on ovarian sympathetic innervation in rats with steroid-induced PCO. To address this question, we studied the mRNA expression and protein amount and distribution of the sympathetic markers α_1a_-, α_1b_-, α_1d_-, and β_2_-AR, and of p75^NTR^.

## Materials and methods

### Animals

Thirty-two virgin adult cycling Wistar Kyoto rats (Möllegaard, Denmark) weighing 205-230g were housed four to a cage at a controlled temperature of 22°C with a 12-h light:12-h dark cycle for at least 1 week before and throughout the experimental periods. The rats had free access to pelleted food and tap water. Sixteen rats, those in the two *PCO groups *described below, were each given a single i.m. injection of 4 mg EV (Riedeldehaen, Germany) in 0.2 ml oil, to induce well-defined PCO [7,18]. Sixteen rats, those in the two *Oil groups *described below, received a single i.m. injection of 0.2 ml oil (arachidis oleum, Apoteket AB, Umeå, Sweden) only. Thirty to thirty-three days after i.m. injection of EV, i.e. 2 days after the last EA treatment, the rats was killed by decapitation. The injections and the finalizing of the experiment was done independent of cycle day [7,18]. The experiments were carried out according to the principles and procedures outlined in the National Institute of Health (NIH) Guide for the Care and Use of Laboratory Animals and were approved by the local animal ethics committee at Göteborg University, Göteborg, Sweden

### Electro-acupuncture treatment

The rats were divided into four experimental groups: i) an Oil group (*control*, n = 8), ii) an Oil group receiving EA (*EA*, n = 8), iii) a PCO group (*PCO*, n = 8), and iv) a PCO group receiving EA (*PCO+EA*, n = 8).

**Figure 1:**
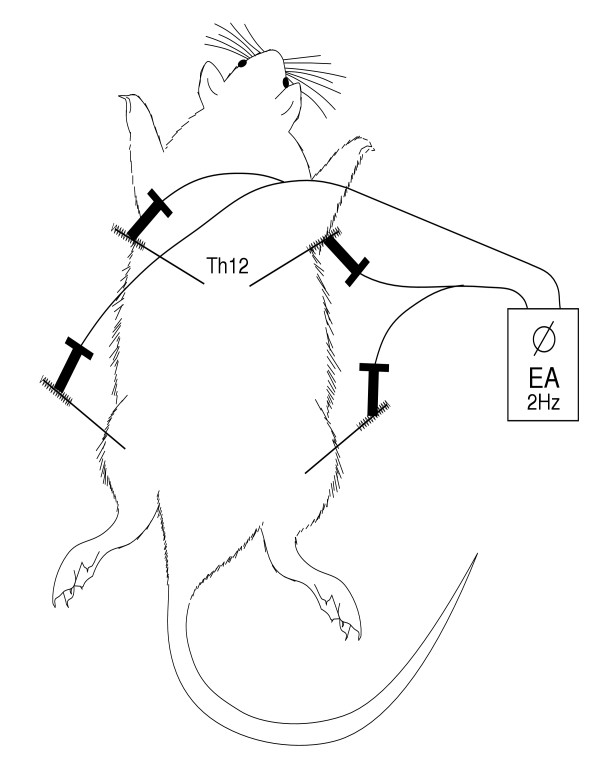
***Schematic drawing of the placement and stimulation of the acupuncture needles***. Two needles were placed bilaterally in m. erector spinae at the level of Th12 and two were placed in m. quadriceps bilaterally. The needles were then attached to an electrical stimulator for electro-acupuncture (EA) treatment. Reprinted with permission from Biol Reprod (2000) 63:1507-1513.

All groups were anaesthetized for 25 minutes on 12 occasions as described below. The *EA *and the *PCO*+*EA *groups received EA every second day during anesthesia. The *EA *and the *PCO*+*EA *group underwent the first EA treatment 2 days after the EV injection. The points chosen for stimulation were bilateral in the mm. biceps femoris and erector spinae, in somatic segments corresponding to the innervation of the ovaries (Figure 1). The needles (Hegu: Hegu AB, Landsbro, Sweden) were inserted to depths of 0.5-0.8 cm and then attached bilaterally to an electrical stimulator (CEFAR ACUS 4, Cefar, Lund, Sweden). The points were electrically stimulated with a low burst frequency of 2 Hz; each pulse had a duration of 180 μsec, a burst length of 0.1 sec, and a burst frequency of 80 Hz. The intensity (1.5-2 mA) was adjusted until local muscle contractions were observed to reflect the activation of muscle-nerve afferents (A-delta fibers and possibly C fibers). The location and type of stimulation were the same in all rats.

### Anaesthetization

During each treatment, all rats were anaesthetized superficially with an intraperitoneal (i.p.) injection of a mixture of Ketamin (50 mg/kg; PARKE-DAVIS, Warner Lambert Nordic AB, Solna, Sweden) and Rompun (20 mg/kg; Bayer, Bayer AG, Leverkusen, Germany). On day 30 after the i.m. injection of EV, the rats was decapitated, that is, 1-2 days after the last EA treatment.

### Tissues

At the completion of the experiment, the ovaries were quickly dissected on dry ice. One ovary was divided in two pieces, weighed, and snap frozen in liquid nitrogen and stored at -80°C until extraction. The second ovary was fixed in buffered 4% formaldehyde for at least 24 hours in preparation for AR, and p75^NTR ^immunohistochemistry.

### Real-Time PCR for adrenoceptors

Total RNA from the ovary was extracted using RNeasy Mini kits (Qiagen, Hilden, Germany). First-strand cDNA was synthesized from 1 μg of total RNA with TaqMan reverse transcription reagents (Applied Biosystems., Foster City, CA). Each 100 μl RT-PCR reaction contained 1 μg of total, 1X TaqMan RT buffer, 5 mM MgCl2, 2.5 mM random hexamers, 1 mM dNTP, 0.4 U/ml RNase inhibitor, and 1.25 U/ ml Multiscribe RT (PE Applied Biosystems, Foster City, CA, USA). Reverse transcription was carried out in a PTC-200 PCR system (MJ Research., Boston, MA, USA) at 25°C for 10 min, 48°C for 30 min and 95°C for 5 min.

The polymerase chain reaction (PCR) analysis was performed using the ABI Prism 7700 Sequence Detection System (PE Applied Biosystems, Stockholm, Sweden) and FAM-labeled probe specific for α_1a_-AR (Rn00567876m1), α_1b_-AR (ADRA A1B-EX 152027A02), α_1d_-AR (Rn00577931ml), and β_2_-AR (Rn00560650s1) (PE Applied Biosystems). Designed primers and a VIC-labeled probe for Glyceraldehyde-3-phosphate dehydrogenase (GAPDH) (NM_031144) were included in the reactions as an internal standard. cDNA was amplified under the following conditions: 1 cycle at 50°C for 2 min and 95°C for 10 min, followed by 40 cycles at 95°C for 15 s and 60°C for 1 min. The amount of mRNA of each gene was calculated using the standard curve method (following the instructions in User Bulletin no. 2, PE Applied Biosystems) and adjusted for the expression of GAPDH.

### Reverse Transcriptase-PCR-ELISA for p75^NTR^

The expression of p75^NTR^-mRNA was evaluated using the reverse transcriptase (RT)-PCR enzyme-linked immunosorbent assay (ELISA) protocol, exactly as previously described by Tirassa and co-workers [24]. Total RNA was extracted from the ovaries using the method of Chomczynski and Sacchi [25] as modified in the TRIzol Kit (Invitrogen AB, Lidingö, Sweden). Complementary DNA was synthesized from 1 μg of total RNA using 250 ng Oligo (dT)_12–18 _primer and 200 Units of M-MLV RT (Promega Italia, Milan, Italy) in 20 μl of total volume reaction. p75 and GAPDH genes were co-amplified in a single-tube PCR reaction (35 cycles: 1 min at 95°C; 1 min at 55°C; 2 min at 72°C) using 5'-biotinylated specific primers to generate biotinylated PCR products detectable by digoxygenin-labeled probes in an immuno-enzymatic assay. Primer/probe sequences are as follows: p75^NTR ^biotinylated forward: ^5'^CGTGTT CTCCTGCCAGGACA^3'^; p75^NTR ^reverse: ^5'^GAGATGCCACTGTCGCTGTG^3'^; p75^NTR ^digoxygenin-labeled probe: ^5'^ACAGCAGCCAAGATGGAGCAATAGACAGG^3'^; GAPDH biotinylated forward: ^5'^CACCACCATGGAGAAGGCC^3'^; GAPDH reverse: ^5'^GATGGATGCCTTGGCCAGG^3'^; GAPDH digoxygenin-labeled probe: ^5'^ACAATCTTGAGTGAGTTGTCATATTTCTCG^3'^. The amount of amplified products was measured at an optical density (O.D.) of 450/690 nm (O.D. 450/690) using a Dynatech ELISA Reader 5000. A GAPDH level of O.D. 450/690 was used to normalise the relative differences in sample size, differences in the integrity of the individual RNA, and variations in RT efficiency. For exact methodological details see Tirassa *et al*. [24].

### Immunohistochemistry for adrenoceptors and p75^NTR^

Commercially available antibodies were used to detect α_1a_-AR (α_1a_-AR [C-19]: *sc-1477*, Santa Cruz, California, USA), α_1b_-AR (α_1b_-AR [C-18]: *sc-1476*, Santa Cruz, California, USA), α_1d_-AR (α_1d_-AR [H-142]: *sc-10721*, Santa Cruz, California, USA), and β_2_-AR (β_2_-AR [M-20]: *sc-1570*, Santa Cruz, California, USA) by immunohistochemistry. The monoclonal antibody anti-p75^NTR ^(clone 192) [26] was produced and purified in our laboratory.

Serial, 15-μm thick sections of each ovary were cut with a cryostat and processed for immunohistochemistry. Briefly, sections were blocked with a 10 minutes incubation in 3% hydrogen peroxide and 10% methanol in PBS containing 0.1% Triton X-100 (PBST), followed by a 30 minutes incubation in 10% normal serum dissolved in PBST. Then, sections were incubated overnight at 4°C with the primary antibody diluted in PBST (rabbit and goat anti-ARs: 5 μg/ml; monoclonal anti p75^NTR^: 1 μg/ml). Sections were then incubated with biotinylated anti-rabbit IgG (α_1d_- and β_2_-AR), anti-goat IgG (α_1a_- and α_1b_-AR) or anti-mouse IgG (p75^NTR^) antibodies (all of them from Vector Lab. Inc., Burlingame, CA, and used according to manufacturer instructions at 1:300 dilution) diluted in PBST. Diaminobenzidine was used to detect the immuno-complex. To assess staining specificity, sections were incubated with non-specific rabbit, goat or mouse IgG (Zymed Lab Inc, San Francisco, Ca) and used as controls. Immunostained sections were evaluated under the Nikon Eclypse E600 microscope equipped with the Nikon DMX 1200 digital camera connected to a PC computer. Sections were coded, and positive cells were counted in 10 sections coming from 5 different ovaries (i.e. 2 sections per ovary) per experimental group. Cell count was carried out using the image processing and analysis program Nikon-Lucia, and measurements were standardized between the experimental groups using the same calibration system and threshold (see below). The number of immunoreactive cells (mean ± SEM) was determined in 20× magnification images over an image area of 40000 μm^2^. Five non-overlapping areas per section were counted. Since the image analyzer determines the optical density of immunoreactions using a grey scale thresholding operation, measurements were standardized between groups using the following criteria: 1) all measurements were conducted after the same calibration of the image analysis system, 2) thresholding was carried out to the same value for each image, 3) the grey scale was calibrated to a range of 25-150 arbitrary units. Objects with higher or lower grey levels were not considered. A morphological program, which selects only cell bodies – but not small fragments or cells that do not have a complete soma – was also used to quantify immunopositive cells.

### Statistical analyses

All statistical evaluations were performed using the Stat View package for Macintosh (Abacus Concepts Inc., Berkeley, CA, USA). mRNA expression and immunopositive cell counts of α_1_- and β_2_-ARs, and p75^NTR ^in the ovaries were evaluated using one-way analysis of variance (ANOVA), and the groups were tested using multiple comparisons with the correction of Fisher PSD. All results are reported as a mean ± standard error of the mean (SEM). A p-value less than 0.05 was considered significant.

## Results

### Ovarian expression and distribution of α_1a-AR_

**Figure 2:**
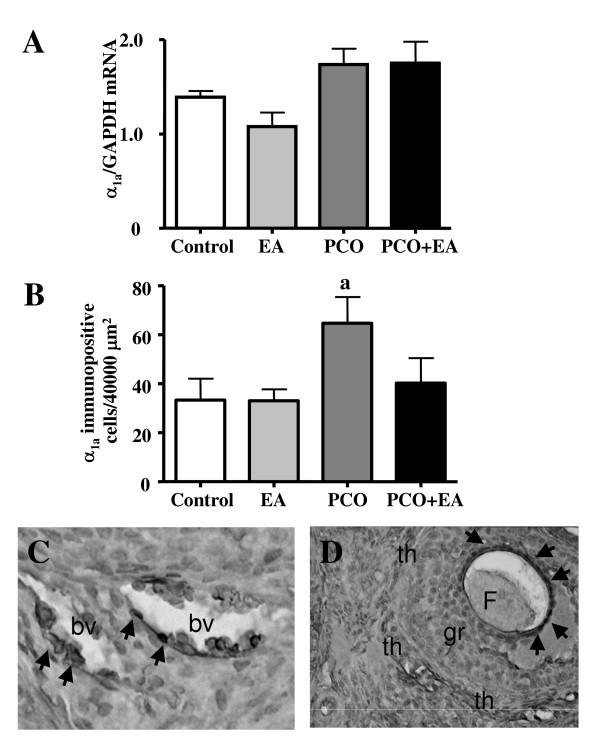
***Ovarian α_1a_-AR mRNA and protein expression***. As shown in panel A, no significant differences were found in α_1a_-AR mRNA expression between the control, EA, and PCO groups. Values are given as means ± SEMs normalized to GAPDH. The results of α_1a_-AR immunopositive cell count is shown in panel B. Values are given as means ± SEMs. Immunostaining revealed that ovarian α_1a_-AR protein is expressed in *control group *ovaries. EA treatments did not affect the number of immunopositive cells in the ovaries of control rats. PCO ovaries had significantly higher amounts of α_1a_-AR protein compared with control ovaries. EA treatments in PCO rats decreased α_1a_-AR protein immunoreactivity when compared with untreated PCO rats. ^a^p < 0.05 vs control group. ^b^p < 0.05 vs PCO group. Representative pictures showing the distribution of α_1a_-AR positive cells in the ovaries of the experimental groups are showed in Panel: C-D. Immunostained cells (arrows) were localized around blood vessels (C) and in the granulosa cells of an early antral follicle (D) and corpora lutea. F: Follicle; bv: blood vessel; gr: granulose cells; th: thecal layer. Magnification C: ×400; D: ×200.

The mRNA expression of α_1a_-AR in the ovary was unaltered in the *PCO group *compared to the *control group*. Repeated low-frequency EA treatments did not affect the mRNA expression of α_1a_-AR in the *EA *or the *PCO*+*EA groups *(Figure 2A).

Significantly higher number of immunopositive cells of α_1a_-AR was found in the *PCO group *compared with the *control group*. EA treatments prevented the increase in α_1a_-AR protein immunoreactivity, since the number of immunostained cells in the *PCO+EA group *was not different from the *control group*. EA treatments did not affect the number of immunopositive cells in the ovaries of control rats. Immunohistochemical analysis on serial sections showed that α_1a_-AR protein (Figure 2C and 2D) was expressed mainly around blood vessels and granulosa regions.

### Ovarian expression and distribution of α_1b_-AR

**Figure 3:**
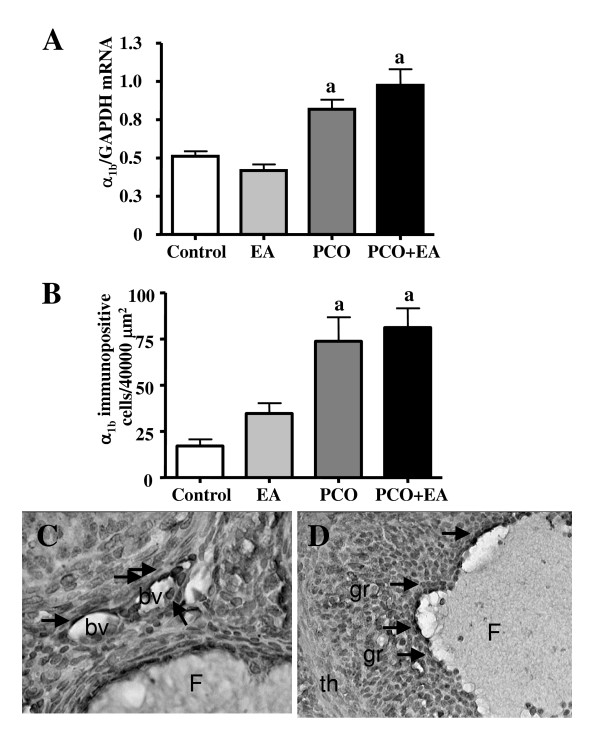
***Ovarian α_1b_-AR mRNA and protein expression***. As shown in panel A, no significant differences were found in α_1b_-AR mRNA between the *control *and *EA groups*. A significant increase in ovarian α_1b_-AR mRNA was found in the *PCO *group when compared to controls. No differences were found between the *PCO*+*EA *group and the *PCO group*. Values are given as means ± SEMs normalized to GAPDH. ^a^p < 0.05 vs control group. EA treatments did not affect the amount of α_1b_-AR immunopositive cells in the *EA group*(panel B). Significantly higher number of immunopositive cells of α_1b_-AR was found in the ovaries of *PCO *rats. EA treatments did not affect the amount or distribution of α_1b_-AR protein in PCO ovaries. Values are given as means ± SEM. ^a^p < 0.05 vs control group. Representative pictures showing the distribution of α_1b_-AR positive cells in the ovaries of the experimental groups are showed in Panels C-D. Immunostained cells (arrows) were localized around blood vessels (C) and in the granulosa cells of mature follicles (D). F: Follicle; bv: blood vessel; gr: granulose cells; th: thecal layer. Magnification C: ×400; D: ×200.

The mRNA expression of α_1b_-AR in the ovary was significantly higher in the *PCO group *than in the *control *and *EA group*. The mRNA expression of α_1b_-AR was significantly higher in the *PCO*+*EA group *compared to the two control groups but not to the *PCO group *(Figure 3A).

As illustrated in Figure 3B, significantly higher number of immunopositive cells of α_1b_-AR was found in the ovaries of *PCO *and *PCO*+*EA *rats when compared to *controls*. EA treatments did not affect the amount or distribution of α_1b_-AR protein in the *EA *and the *PCO groups*. As shown in Figure 3C–D, α_1b_-AR protein was located around blood vessels and in the granulose region of mature follicles.

### Ovarian expression and distribution of α_1d_-AR

**Figure 4:**
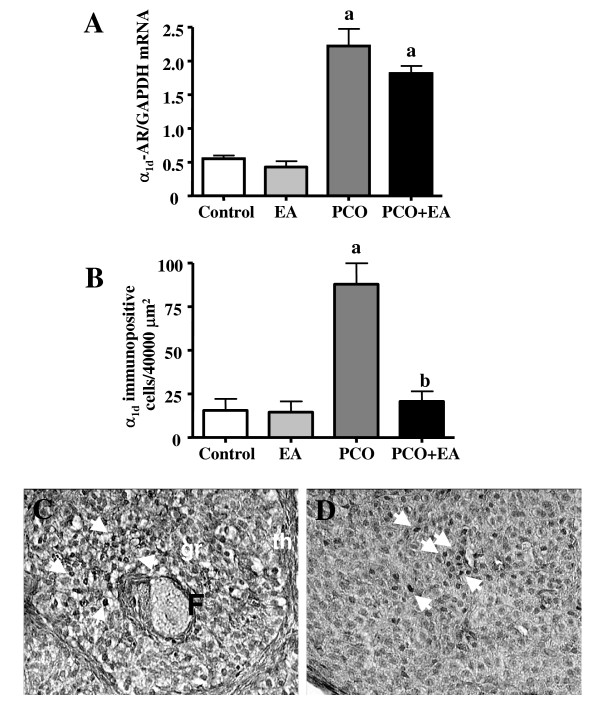
***Ovarian α_1d_-AR mRNA and protein expression***. As shown in panel A, α_1d_-AR mRNA expression was significantly higher in the *PCO group *than in *control *and *EA group*. EA significantly decreased the mRNA expression in the *PCO*+*EA group *compared with the *PCO group*. Values are given as means ± SEMs normalized to GAPDH. ^a^p < 0.05 vs control group. The α_1d_-AR immunopositive cell number (Panel B) was not affect in the *EA group *when compared to *control group*. Significantly higher number of immunopositive cells of α_1d_-AR was found in the *PCO group *compared with the *control group*. EA treatment significantly decreased the number of immunopositive cells in the *PCO*+*EA group*. Values are given as means ± SEMs. ^a^p < 0.05 vs control group. ^b^p < 0.05 vs PCO group. Representative pictures showing ovarian distribution of α_1d_-AR expressing cells (pointed by arrows) are showed in Panels C-D. The α_1d_-AR was found expressed in the granulosa cells of healthy follicles (C) and corpora lutea (D) and around blood vessels (not shown) in all of the experimental groups. F: Follicle; gr: granulose cells; th: thecal layer. Magnification C-D: ×200.

The mRNA expression of α_1d_-AR in the ovary was significantly higher in the *PCO group *than in the *control group*. The mRNA expression of α_1d_-AR in the *PCO*+*EA *was not different from that in the *PCO group *(Figure 4A).

As illustrated in Figure 4B, significantly higher number of immunopositive cells of α_1d_-AR was found in the *PCO group *compared with the *control group*. EA treatment significantly decreased the number of immunopositive cells in the *PCO+EA group*. The α_1d_-AR immunopositive cell number was not affected in the *EA group *when compared to *control group*. As shown in Figure 4C–D, the α_1d_-AR was found expressed in the granulosa cells of healthy follicles and corpora lutea, and around blood vessels (not shown) in all of the experimental groups.

### Ovarian expression and distribution of β_2_-AR

**Figure 5:**
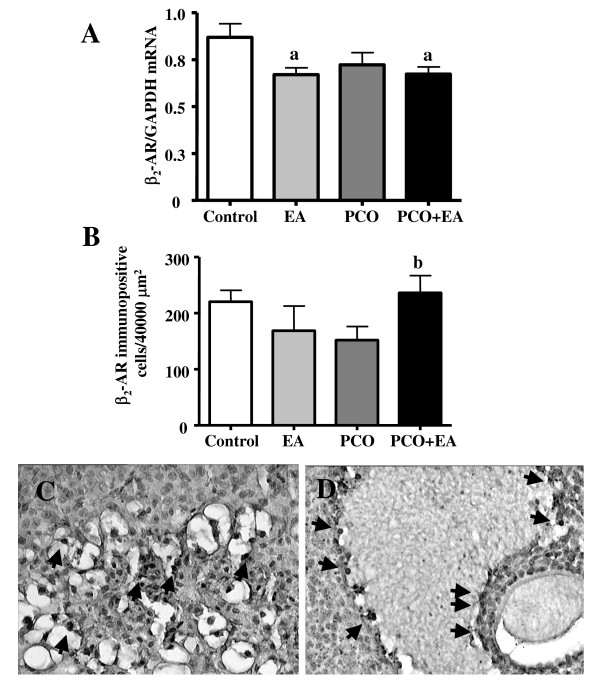
***Ovarian *β_*2*_-*AR mRNA and protein expression***. The expression of β_2_-AR mRNA in the ovary (panel A) in the *PCO group *was lower compared to the *control group*. β_2_-AR mRNA was unaltered in the *PCO*+*EA group *when compared to *control*. Values are given as means ± SEMs normalized to GAPDH. ^a^p < 0.05 vs control group. β_2_-AR immunopositive cell number (Panel B) in control ovaries was unchanged by EA treatments. No difference in number of β_2_-AR immunoreactive cells was found in PCO ovaries, while EA treatments in PCO rats significantly increase the amount of β_2_-AR immunostained cells when compared to PCO group. Values are given as means ± SEMs. ^b^p < 0.05 vs PCO group. Representative pictures of stained cells (some of them pointed by black arrows) are showed in Panels C- D. The β_2_-AR was found expressed in degenerating corpora lutea (C) and follicles (D) in all of the experimental groups. Magnification C: ×400; D: ×200.

The mRNA expression of β_2_-AR in the ovary of the *PCO group *was not changed, when compared to the *control group*. β_2_-AR mRNA was significantly lower both in the *EA group *and in the *PCO+EA group *when compared to the *control group *(Figure 5A).

No difference in number of β_2_-AR immunoreactive cells was found in PCO ovaries (Figure 5B), while EA treatments in PCO rats (*PCO+EA group*) significantly increase the amount of β_2_-AR immunostained cells when compared to *PCO group*. β_2_-AR immunopositive cell number in control ovaries was unchanged by EA treatments. The β_2_-AR was found expressed in degenerating corpora lutea (Figure 5C) and follicles (Figure 5D) in all of the experimental groups.

### Ovarian expression and distribution of p75^NTR^

**Figure 6:**
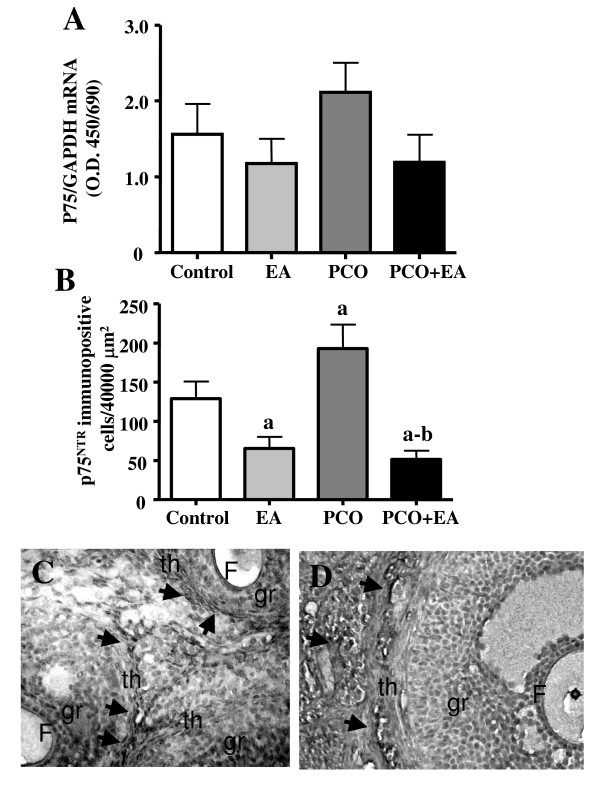
***Ovarian p75^NTR ^mRNA and protein expression***. As shown in panel A, ovarian p75^NTR ^mRNA was found unchanged in the *PCO group *compared to the *control *and *EA group*. Low-frequency EA treatments did not significantly affect ovarian p75^NTR^mRNA expression in the *EA, PCO and PCO+EA groups*. Values are given as means ± SEMs normalized to GAPDH. As shown in panel B, the number of ovarian p75^NTR ^immunopositive cells was significantly decreased in the *EA group *when compared to *controls*. The number of p75^NTR^-stained cells in the *PCO group *was significantly higher than in controls, while repeated EA treatments greatly decreased p75^NTR ^protein immunoreactivity in the *PCO*+*EA group*. Values are given as means ± SEMs. ^a^p < 0.05 vs control group. ^b^p < 0.05 vs PCO group. Representative pictures of stained cells (pointed by black arrows) are showed in Panels C-D. The p75NTR was found expressed in the thecal layer of healthy follicles (C) and in the stromal region (D) in all of the experimental groups. Magnification C-D: ×200.

The p75^NTR ^mRNA expression in the ovary (Figure 6A) was unchanged in the *PCO group *compared to the *control group*. Low-frequency EA treatments did not affect ovarian p75^NTR ^mRNA expression in the *PCO+EA *group compared to the *PCO group*, and did not differ from the *control group*.

As shown in Figure 6B, the number of p75^NTR ^-stained cells in the *PCO group *was significantly higher than in controls, while repeated EA treatments significantly decreased p75^NTR ^protein immunoreactivity in the *PCO+EA group*. The number of ovarian p75^NTR ^immunopositive cells was significantly lower in the *EA group *when compared to *controls*. As shown in Figure 6C–D, ovarian p75^NTR ^expressing cells were distributed mainly around the follicles in the theca layers, with some immunoreactivity also spread in the ovarian stroma (Figure 6D).

## Discussion

The aim of the present study was to investigate whether repeated low-frequency EA treatments modulate the expression of mRNA and the amount and distribution of proteins of α_1_-, and β_2_-ARs, and p75^NTR ^in rats with steroid-induced PCO. The results of this study demonstrated that i.m. EV injections result in significantly higher mRNA expression of ovarian α_1b_- and α_1d_-AR in PCO rats compared to control rats. EV-induced PCO induced a significantly higher amount of immunostained cells for α_1a_-, α_1b_- and α_1d _proteins, that was prevented by repeated low-frequency EA treatments, except for α_1b_-AR. The EA treatment also induced an increase of β_2 _-AR protein in the EV-injected rats.

It has been suggested that high sympathetic drive to the ovary might be important in both EV-induced PCO in rats and PCOS in humans [4,5,9,10,27]. Clinical studies show that women with PCOS temporarily recover normal ovarian function after bilateral wedge resection or ovarian drilling, which partially denervates the ovary [28]. These observations suggest that the ovarian nerves might be involved in the successful outcome of bilateral wedge resection and ovarian drilling. Current pharmacological treatment using clomiphene citrate is the first-line treatment for ovulation induction in women with PCOS [29]. This is effective, but side-effects such as super ovulation are quite common [30]. There is a clear need to identify new therapeutical approaches – including non-pharmacological strategies – to reduce or replace drug intervention.

That EA may reduce hyperactivity in the ovarian peripheral sympathetic nerve fibers is consistent with the theory that EA could modulate sensory, motor, and autonomic outflow at the segmental level [16]. It has also been shown that EA activates higher control systems, resulting in the release of a number of neuropeptides that are important in the modulation of central and segmental autonomic outflow and of the HPO axis [16,31]. We have recently shown that repeated low-frequency EA induces regular ovulations in more than one-third of women with PCOS and normalizes endocrine and neuroendocrine parameters without any negative side-effects [17]. The effects of repeated low-frequency EA were then attributed to an inhibition of hyperactivity in the sympathetic nervous system [16,32]. The present study further indicates that EA is effective in preventing EV-induced dysregulation of ovarian sympathetic markers.

Increased peripheral sympathetic outflow in rats with steroid-induced PCO is evidenced by increased releases of NE, higher concentrations of NE in the ovary, and a reduced number of β_2_-AR in the ovarian compartment receiving catecholaminergic innervation [8,9]. The role of β_2_-AR in ovarian physiology and pathophysiology has been related to the regulation of ovarian steroidogenesis [8]. Transection of the superior ovarian nerve in steroid-induced PCO reduces the steroid response, raises β_2_-AR expression to normal levels, and restores estrus cyclicity and ovulation [8]. Thus the disturbances in steroid production – at least in the present rat PCO model – might be secondary to the elevated adrenergic control over ovarian steroidogenesis mediated by β_2_-AR. Interestingly, repeated treatments of low-frequency EA induced an increase of β_2_-AR protein in EV-injected rats, and this is in accordance with the hypothesis that EA down-regulates the activity in the sympathetic nervous system. On the other hand, the mRNA expression of β_2_-AR was decreased in the EA and in the PCO+EA group compared with the control group. One plausible explanation for the discrepancy between the mRNA and protein levels might be an unbalanced turn over between β_2_-AR mRNA and protein. Thus the lower level of mRNA, compared to those of the protein, could reflect its utilization for protein synthesis, not balanced by an appropriate mRNA replacement. Such a mechanism could also reflect different regulation levels for our treatments, that could act on protein production separately at both the gene transcription and protein synthesis. Further studies are necessary to clarify this mechanism(s).

The functional significance of the different α_1_-ARs in the ovary of PCO rats has not been clearly identified. The function of these types of ARs has traditionally been characterized in ovarian physiology as being primarily related to the regulation of ovarian blood flow. Interestingly, α_1_-agonist stimulation has recently been shown to modulate the progesterone release in cultured granulose cells by potentiation of vasoactive intestinal peptide (VIP) and Pituitary Adenylate Cyclase-Activating Polypeptide (PACAP) [33]. In a recent study, for the first time to our knowledge, we have shown that the expression of all α_1_-AR subtypes at both the mRNA and the protein level are up-regulated at an early (30 day) and at a late (60 day) stage after EV injection [14]. Thus it can be inferred that α_1_-ARs participate not only in the physiological regulation of progesterone from the normal rat ovary [33], but also most probably in the up-regulation of progesterone release described in the EV-induced PCO ovary [8]. Furthermore, the dysregulated α_1_-ARs can be related to high sympathetic activity in the ovaries of PCO rats [8]. Indeed, we found that repeated treatments of low-frequency EA prevents the dysregulation of α_1a_-AR and α_1d_-AR protein in rats with steroid-induced PCO that evidence the effectiveness of EA in reducing the sympathetic drive to the ovary. The mRNA expression of α_1d_-AR was normalized by low-frequency EA, but not that of α_1a_- and α_1b_-AR. Again, the discrepancy between the mRNA expression and protein levels might be an unbalanced turn over between α_1a_- and α_1b_-ARs mRNA and protein, thus the mRNA that is engaged in the protein translation is not replaced completely, while this is not the case for α_1d_-AR. These results indicate that a mechanism linked to ovarian presence and function of ARs could be active in this context.

The present results are in line with the results of a recent study demonstrating that low-frequency EA increased blood flow and decreased sympathetic activity in the ovary [22,23]. These observations led to the hypothesis that the effects of low-frequency EA on ovarian blood flow were mediated by α_1_-ARs [22], and this is in line with another recent study by Uchida et al. [12] which suggested that the regulation of ovarian blood flow via sensory stimulation is mediated by α_1_-ARs evidenced by blocking α_1_-ARs.

In the present study, we also demonstrate that repeated treatments of low-frequency EA maintains p75^NTR ^mRNA and protein amount at basal levels in PCO animals. This agrees with results of our previous studies [18,20]. It is known that p75^NTR ^aids the development of specific populations of sympathetic neurons [34], and that this receptor is responsible for the responsiveness of adult sympathetic neurons to target-derived NGF [35]. The evidence that EV injection in adult rats increases the intraovarian synthesis of both NGF and p75^NTR ^[10] suggests a possible functional link between PCO and the NGF/NGF receptor system. Interestingly, these changes were accompanied by selective activation of noradrenergic neurons projecting to the ovary. The activation of the sympathetic nervous system after EV injection has been evidenced by enhanced TH activity in the ovaries of PCO rats [9] and by increase of TH mRNA expression in the catecholaminergic cells of the celiac ganglion selectively projecting to the ovaries [10]. Furthermore, intraovarian blockade of NGF and p75^NTR ^resulted in decreased p75^NTR ^synthesis by ovarian theca cells and restored estrous cyclicity and ovulatory capacity in EV-injected rats [10]. Thus, one possible mechanism underlying the effect of low-frequency EA on sympathetic tone might be decreased p75^NTR^-mediated sympathetic responsiveness to NGF action. That EA counteracted the EV-induced increase in ovarian expression and amount of p75^NTR ^supports this hypothesis.

Interestingly, CRF a principal neurohormone in the control of the hypothalamus-pituitary-adrenal (HPA) axis, has been shown to be increased in both the median eminence and in the ovary in rats with steroid-induced PCO [19]. In the same study, repeated low-frequency EA restored the increased CRF concentrations indicating that peripheral CRF and the HPA axis plays a crucial role in the regulation of ovarian function in steroid-induced PCO [19]. These data, together with the present one, suggest that EA could act as a modulator of the central control over sympathetic output in rats with steroid-induced PCO. Further studies are necessary to clarify this point.

In conclusion, the present study shows that EA prevented most of the EV-induced changes in ovarian ARs. Furthermore, EA was able to counteract the EV-induced up regulation of p75^NTR^, probably by normalizing the sympathetic ovarian response to NGF action. Our data indicate the effectiveness of EA in the regulation of ovarian responsiveness to sympathetic inputs and depict a possible complementary therapeutic approach to preventing and/or overcoming sympathetic-related anovulation in women with PCOS.

## Authors' contributions

LM participated in the design of the study, carried out part of the animal preparation, performed RT-PCR and immunohistochemical analyses, performed the statistical analysis and drafted the manuscript. TL, AH and LA participated in the design of the study and in writing the manuscript. ES-V participated in the design of the study, carried out part of the animal preparation and drafted the manuscript. All authors read and approved the final manuscript.
